# Supplementary material for: Are carbon emissions trading and green financial instruments synergistic? -Comprehensive quantitative research based on content analysis
Source: PLoS One. 2024 Mar 7;19(3):e0298601. doi: 10.1371/journal.pone.0298601 (PMC10919601; doi:10.1371/journal.pone.0298601)
Supplement: S1 Data — (ZIP) [file pone.0298601.s001.zip › Data/地级市Z_F工作报告.zip!/╣π╢1⁄2/╔╟═╖╩╨/└┤╫╘╣1⁄2╓┌║┼í╛┬φ┐╦╩2╛▌═°í┐_2011.docx]

各位代表：

　　我代表市人民政府向大会报告工作，请予审议，并请政协各位委员和其他列席人员提出意见。

　　一、“十一五”时期工作回顾

　　“十一五”时期是汕头经济社会持续平稳较快发展的时期。在省委、省政府和市委的正确领导下，市政府坚持以科学发展观为指导，全面贯彻落实“三年打基础、五年大变化、十年大发展”的决策部署，顺利完成“十一五”规划确定的主要目标和任务。

　　——经济综合实力迈上新台阶。2010年全市生产总值1203.25亿元，同比增长13.7%，五年间年均增长11.9%。人均生产总值23274元，年均增长10.9%，实现比2000年翻一番的目标。规模以上工业总产值、全社会固定资产投资额、社会消费品零售总额、外贸进出口总额分别达到1887.42亿元、361.68亿元、830.41亿元、73.64亿美元，年均增长16.4%、18.4%、19.2%、8.2%。经济效益持续改善，地方财政一般预算收入72.65亿元，同比增长24.1%，年均增长19.8%，占GDP比重为6%，比“十五”期末提高1.4个百分点。预期完成“十一五”节能减排目标任务。

　　——产业结构不断优化。工业产业向集群化、高端化发展，企业自主创新能力明显提高，科技指标和名牌数量居全省前列，荣获“国家知识产权工作示范城市”称号。大力扶持企业上市，2010年新增上市企业9家，合计16家，总数列全省第3位。服务业稳步发展，港口货物吞吐量3510万吨、集装箱吞吐量93.5万标箱、机场旅客吞吐量172.8万人次，年均增长15.1%、20.5%、14.4%，汽车、住房、教育、旅游等成为新的消费热点。加快特色农产品基地和现代农业园区建设，推进农业产业化经营，农业效益明显提高。

　　——城乡发展水平稳步提升。强化规划引领功能，完成一批涉及全局的重大规划和专项规划，城市建成区面积达到175平方公里。克服征地拆迁和财政资金紧缺等困难，金凤路桥建成通车，改造天山北路、黄山路等一批“断头路”、“水浸街”，实施龙湖沟、新河沟等河沟环境整治。调整城市管理体制机制和事权财权，着力构建城市管理长效机制，开通城市管理服务热线，综合整治市容市貌，城市面貌有所改观。加大园林绿化建设力度，2010年荣获“国家园林城市”称号。完成中心城区82个“城中村”及涉农社区供水直抄到户改造。新农村建设有序推进，村容村貌整治和村庄规划得到加强，建成农村硬底化公路1018公里，解决农村50万人的饮水安全问题。

　　——基础设施建设取得新进展。强力推进厦深铁路、汕头至揭阳高速公路、南澳大桥等重大项目建设，建成广澳港区5万吨级泊位和5万吨级航道。加快推进汕头至揭西等高速公路和疏港铁路等重点项目前期工作。华能海门电厂1、2号机组和500千伏送出工程等一批能源项目投产。完成牛田洋海堤、秋风岭水库等一批江海堤围、水闸和水库除险加固工程。一批生活污水处理设施和生活垃圾无害化处理设施建成投入使用，五年新增城市生活污水处理能力44.7万吨/日，全市达58.7万吨/日。产业转移工业园获省认定为示范园区，潮南产业转移工业园完成首期基础设施建设，南山湾科技园、濠江电子电路工业园加快建设。

　　——开放合作实现新突破。2010年外贸出口总额49.35亿美元，同比增长22.9%，年均增长9.2%。2010年实际吸收外商直接投资2.56亿美元，同比增长25.2%，五年累计实际吸收外商直接投资9.6亿美元。我市企业到境外投资设立加工贸易企业、合作开发资源步伐加快。口岸通关环境进一步改善，开通国际集装箱航线11条，广澳港区通过国家一类口岸验收；汕头机场获准对东南亚国家和港澳地区航空公司飞机开放。积极参与海峡西岸经济区建设，深化对台合作，实现对台海上货运直航和对台旅游包船直航，设立台湾农民创业园，率先建设粤台经贸合作试验区。成功举办粤东侨博会、“山洽会”、国际潮青联谊年会。全面完成对口援建草坡乡任务。

　　——各项改革进一步深化。建立市行政服务中心，进驻单位56个、进驻事项1034项，实施市级工程建设项目并联审批，提高审批效率。完成第三、四轮行政审批改革，削减审批事项61.6%，46个单位461项行政审批事项纳入行政电子监察系统，实现省、市、区县和部分街道（镇）四级联网。开展“百人服务团”活动，解决答复企业反映问题834项。民营经济发展环境不断优化，2010年民营经济实现增加值713.52亿元，同比增长15%。国有企业改革稳步推进，完成172家企业改革重组。汕头商行重组取得重大进展，汕特农联社改革向重组方向发展。

　　——社会事业全面发展。人民生活水平稳步提高，2010年中心城区居民人均可支配收入15179元，农村居民人均纯收入6518元，年均增长7.4%、7.2%。全面实施城乡免费义务教育，改造中小学校舍63.6万平方米；加快普及高中阶段教育，五年新增学位10万个，毛入学率由44.72%提高至85.26%，教育质量进一步提高。妥善解决中小学代课教师问题，录用“代转公”教师4096人。劳动就业形势稳定，城镇登记失业率控制在3.3%以内，五年全市城镇新增就业35万人，农村劳动力转移就业28.73万人。社会保障、社会救助覆盖面进一步扩大，城镇职工基本养老保险、城镇基本医疗覆盖率分别为39.8%、86.6%，新型农村合作医疗制度覆盖所有镇村，参合率达到99%。设立贫困精神病人康复救助基金，建立精神病人防治救助长效机制。加大保障性住房建设力度，五年共筹集廉租公房1752套，发放租赁住房补贴777.85万元。基本解决市区商品房确权发证遗留问题，五年完成登记审核34430套。安全生产取得明显进展，五年全市各类事故死亡总量年均下降8.5%。实施扶持老区山区建设项目48个，扎实推进扶贫开发“双到”工作，帮助4708户贫困户脱贫。文体事业全面繁荣，文化惠民工程扎实推进，市图书馆、文化艺术学校、潮汕侨批文物馆等一批文化设施相继投入使用。文化遗产保护成效显著，对“南澳I号”古沉船进行水下抢救性发掘。《东吴郡主》等一批文艺精品获殊荣。我市运动员获北京奥运会金牌2枚、广州亚运会金牌3枚。人口和计划生育工作稳步推进，2010年全市人口出生率、人口自然增长率分别为9.54％。和4.77％。。第六次全国人口普查工作进展顺利。完成省下达的年度殡葬管理目标任务。外事侨务、妇女儿童、双拥、优抚安置、老龄、慈善和社会福利事业加快发展，统计、粮食、人防、地震、气象、档案、方志、民族、宗教等工作取得新成绩。

　　——政府自身建设得到加强。认真执行法律法规和人大各项决议，自觉接受人大的法律监督、工作监督和人民政协、民主党派的民主监督，办理人大议案（建议）409件，政协提案 852件。大力推进依法行政，提请市人大常委会审议法规草案14项，制定、修改和废止了一批政府规章。启动行政复议委员会试点工作，实施行政复议相对集中审理。建立市、区两级政府应急管理机构，提高突发事件处置能力。开展领导干部大接访活动，推行“民声热线”、网络问政等新举措，认真解决群众反映的热点难点问题。规范市场秩序，建立价格调节基金制度，做好市场价格运行监测预警、调控和监管。

　　各位代表！过去五年，我市经济社会发展取得的成绩，是省委、省政府和市委正确领导的结果，是各级人大及其常委会和人民政协监督支持的结果，是全市人民同心同德、奋力拼搏的结果，也是社会各方面支持和帮助的结果。在此，我代表市人民政府，向全市人民，向中央和省驻汕单位，向驻汕人民解放军、武警官兵、人民警察和各民主党派、人民团体、社会各界人士表示崇高的敬意！向关心和支持汕头建设的港澳台同胞、海外侨胞和国际友人表示衷心的感谢！

　　我们也清醒地认识到，我市经济社会发展仍存在不少困难和问题：产业规划和园区建设滞后，产业发展基础薄弱，经济总量仍然偏小；工业投资增量不足，缺乏大的生产性投资项目，制约发展后劲；财政收支矛盾突出，教育、卫生、社保、就业等社会事业发展任务艰巨；交通秩序、治安形势、市容市貌尚未根本改观，群众反映强烈；政府职能转变不到位，政府执行力亟待增强，发展环境仍需不断优化。对这些问题，我们必须采取更加有力的措施认真予以解决。

　　二、“十二五”时期的奋斗目标

　　“十二五”时期，是我市实现“五年大变化，十年大发展”的关键时期。世界经济调整回升，国内经济发展将步入工业化、城镇化中后期，市场需求潜力巨大；汕头被列入国家主体功能区重点开发区域、海峡西岸经济区南翼中心城市、省建设海洋强省试点城市，为我市融入区域发展与合作迎来难得的机遇。特别是当前汕头广大干部群众不甘落后，求新求快求变的愿望十分强烈，这是我们加快发展、建设幸福汕头的强大动力。与此同时，国际经济形势错综复杂，国家实施稳健货币政策，我市面临着加快经济发展速度和加快经济发展方式转变的双重压力，调整经济结构、实现跨越式发展的任务十分艰巨，保障和改善民生的任务十分繁重。为此，我们必须全面把握形势，全力攻坚克难，努力推进汕头经济社会跨越式发展。

　　根据市委九届十次全会提出的《中共汕头市委关于制定全市国民经济和社会发展第十二个五年规划的建议》，市政府组织编制了《汕头市国民经济和社会发展第十二个五年规划纲要（草案）》，提请本次会议审查批准，明确提出我市“十二五”时期经济社会发展的指导思想和发展目标，到2015年，全市生产总值达到3400亿元以上，五年年均增长20%左右；人均生产总值61100元，年均增长19%左右；地方财政收入达到240亿元，年均增长29%，用于民生事业的支出占一般预算支出80%以上，实现基本公共服务均等化。城乡居民收入普遍较快增加，年均增长20%左右，分别达到37800元、16200元，居民消费价格总指数涨幅年均控制在3%左右，人民生活质量和水平不断提高，广大人民群众同享经济发展和社会文明进步成果，建设幸福汕头初见成效。

　　做好“十二五”工作，必须强化特区意识，注重改革创新，着力解决经济社会发展中的深层次矛盾和问题；必须充分发挥侨乡、海洋、港口、对台优势，走超常规发展路子，努力实现跨越式发展；必须着力提升经济发展质量和效益，坚持在发展中促进转变、在转变中加快发展；必须坚持以人为本，始终把改善民生作为经济发展的出发点和落脚点，加快推进基本公共服务均等化，让发展成果真正惠及全市人民；必须注重协调发展，实现经济社会发展与人口、资源、环境相协调，经济效益、社会效益与生态效益的有机统一。

　　三、2011年工作安排

　　2011年是汕头经济特区建立30周年，也是我市实施“十二五”规划的开局之年。政府工作的总体要求是：全面贯彻落实党的十七届五中全会、中央经济工作会议和省委十届八次全会、市委九届十次全会精神，以科学发展为主题，以建设幸福汕头为目标，强化特区意识，擦亮特区牌子，坚持实施投资拉动，调整经济结构，深化改革创新，着力改善民生，推动经济社会又好又快发展，确保实现“五年大变化”。

　　今年经济社会发展的预期目标是：地区生产总值增长15%，工业增加值增长14%，第三产业增加值增长18%，一般预算收入增长20%，工业总产值增长14%，农业总产值增长3%，批零销售额和住宿餐饮营业额增长23%，外贸出口总额增长18%，外贸进口总额增长25%，全社会固定资产投资额增长38%，实际吸收外商投资总额增长35%，人口自然增长率7.5％。，城镇登记失业率控制在2.5%以内，居民消费价格指数涨幅控制在4%左右，城镇居民人均可支配收入增长12.5%，农民人均纯收入增长13%。单位生产总值综合能耗、二氧化硫排放量和化学需氧量排放量完成省下达的控制目标。

　　为此，要全力以赴做好八方面工作：

　　（一）推进产业转型升级

　　实施新兴产业倍增计划。引进中核集团规划建设“中国锆城”。规划建设澄海创意动漫文化产业基地，加快推进金刚玻璃太阳能生产线、合成云母产业基地、数字化超声仪器生产基地、广东移动粤东区域生产项目、新型环保防伪包装材料项目建设。优化产业空间布局，加快省示范性产业转移园濠江电子电路工业园、潮南产业转移工业园、南山湾科技园和潮阳经济技术开发试验区建设。

　　实施现代服务业提速计划。加快推进红星美凯龙、百脑汇资讯广场、苏宁电器粤东总部等一批大型商业综合项目和美莱顺内衣城、龙湖现代物流园、宝供（澄海）物流城建设。力争汕头商行重组成功并复业经营，汕特农信社启动改革重组，潮阳区、澄海区村镇银行顺利开业，中信银行、东亚银行在汕设立机构。规划建设新会展中心。大力发展旅游业，推进建设广东省旅游综合改革示范市工作。

　　实施传统优势产业提升计划。加强质量强市工作。创新政产学研合作发展模式，促进政府、企业和高等院校、科研机构建立技术联盟和产业联盟。积极推进省市共建玩具礼品产业基地、机械联盟项目、纳米印刷产业化基地建设。构建各类公共服务平台，加快国家玩具质量监督检验中心、中国检验检疫科学研究院（汕头）玩具安全研究中心、粤东云计算中心、潮南粤东纺织服装创新基地建设。继续加强技术创新专业镇建设，鼓励和支持企业创品牌，推动品牌产品企业做强做大。

　　实施海洋综合开发计划。加快海洋综合开发规划编制工作，推进广东省海洋综合开发南澳试验县建设，打造粤东海洋经济区。重点加快华能海门电厂3、4号机组，海门港区粤东煤炭中转基地，海门、云澳中心渔港，达濠一级渔港，南澳海洋风机建设，积极培育海洋生物制药、海洋化工、海水淡化和综合利用等产业。

　　提升现代农业发展水平。重点规划建设澄海莲华花卉、濠江观光农业优势产业带，加强台湾农民创业园招商引资，加快台湾农产品交易中心、丹樱生态旅游农业开发项目和南澳海洋生态种养基地建设。全力推进汕头大围六个应急堤段达标加固工程等重点水利项目建设。

　　狠抓节能减排和生态保护。做好国家环保模范城市复检工作。继续推进重点领域和重点企业节能，坚决打击违法排污行为，加快淘汰落后产能。推进环保基础设施建设，完善龙珠水质净化厂等7座污水处理厂管网配套，完成南区污水处理厂濠江分厂主体工程、澄海区洁源垃圾发电厂项目，开工建设谷饶污水处理厂、南区污水处理厂海门分厂、市雷打石环保电厂，加快中心镇及练江流域污水处理设施建设。积极推进贵屿镇国家循环经济试点、省市共建循环经济工业园建设。严格环保准入，深入推进重点流域区域污染防治，加强饮用水源和生态环境保护，提高环境安全保障水平。

　　（二）全力加快城市化进程

　　突出规划的先导作用。加快推进城市总体规划修编和“一湾两岸”、濠江新城、珠港新城、西片区等一系列重点片区的规划编制工作。超前谋划澄海、潮阳、潮南、南澳的城乡规划，一步到位把规划覆盖到全市城乡，推进城乡规划一体化。

　　加快城市开发建设步伐。引进中信集团加快濠江新城建设，启动苏埃湾过海隧道、安海路等南区重大基础设施建设和土地一级开发。深化与中交集团的战略合作，全力加快东海岸新城建设开发步伐。引进战略合作伙伴，投资建设以高新产业、综合服务业、国际湿地保护区和生态示范区为重点的西部生态新城。加大对闲置土地和国有存量土地的清理整治力度，进一步盘活土地资源。调整珠池港区功能，规划建设珠港新城，重点发展总部经济和高端服务业。抓紧大学路路面改造、潮汕路大修工程，缓解潮汕机场、厦深铁路开通后的交通压力。建设金鸿公路、磊广大道路灯配套工程。

　　加强城市管理。加强宜居城乡建设绩效考核。改造金砂路金砂公园两侧、华山路和珠江路的“水浸街”。推进汕樟北路、泰山路等道路延伸工程。启动西堤路、潮阳路等一批市政道路改造。加快推进磊口大桥等7座公路危桥拓宽改建，确保年底前全部完工。加大“治摩”力度，全面取缔人力三轮车，改善交通秩序。着力创新城市管理体制，推行网格化的城市管理模式，进一步建立健全城市管理长效机制。深入有效开展城市环境综合治理，巩固国家园林城市成果，确保顺利通过国家卫生城市复查。

　　大力推进“三旧”改造。有计划地建设一批具有汕头特色的“三旧”改造示范点，重点启动龙湖工业区创意产业园、海滨文化公园、小公园历史建筑风貌保护区、旧城区西片区、西港－光华片区、乌桥岛片区、梅溪河沿岸等一批旧城改造项目。推进村庄整治，争取每年整治10%的村（居）。

　　（三）深化重点领域改革

　　深化行政管理体制改革。加快推进濠江区大部制改革试点工作，深化市县级机构改革。推进事业单位分类改革和人事制度改革。深化审批制度改革，进一步优化工程建设项目审批流程。加快建设行政审批和公共服务综合信息系统，建成覆盖全市各级政府的电子政务平台，推动网上审批、网上服务、网上办公。创新政府公共服务提供方式，培育发展社会组织，积极推进政府购买公共服务。

　　深化财税和投融资体制改革。注重培植税源，大力推进综合治税工作，强化税收征管。不断完善财政管理体制，调整和优化财政收支结构，扩大公共财政覆盖范围，逐步增加公共服务支出。引入市场竞争机制和资本运营理念，整合盘活各种政府有形资产和无形资产，建立科学、高效、可操作的投融资平台，实现多元化经营城市。加快国企改革，加强国资监管，确保国有资产保值增值。

　　推进统筹城乡综合配套改革。积极争取国家批准汕头经济特区范围扩大到全市，支持我市开展统筹城乡综合配套改革。重点推进城乡一体化发展，争取在土地制度改革、户籍制度改革等方面赋予汕头先行先试试验权，探索新型城市化和城乡一体化发展新路子。稳步推进富县强镇事权改革，扩大区县、镇级政府经济社会管理权限。完善基层社会管理体系，强化城乡自治组织自我管理功能。

　　创新引才用才机制。加快出台吸引培养高层次人才的意见，打破身份、人缘、地域界限，面向海内外吸引优秀高端人才来汕干事创业。建立健全以政府奖励为导向、用人单位和社会力量奖励为主体的人才奖励体系，重奖有突出贡献的人才。建立健全科学的人才评价机制，修订完善来汕高层次人才专业技术职务资格认定制度。强化人才服务保障，建立引进人才“一站式”服务平台，启动人才公寓建设，努力解决家属就业、子女读书、医疗保障和居住等问题。

　　促进民营经济发展上水平。加强和改善服务，优化民营经济发展环境。拓宽民间资本投资领域，鼓励引导民间资本进入基础设施、战略性新兴产业、现代服务业和民生事业。加大政策支持力度，扶持中小企业外贸出口、技术改造和技术创新、创立名牌、上市融资，力争今年有3~5家企业上市。

　　（四）进一步提高开放合作水平

　　深入实施外向带动战略。加强与潮籍华侨新生代的联络沟通，吸引海内外潮人潮商回乡投资兴业，积极打造潮商总部集聚地。不断优化贸易结构，继续组织和支持企业开拓国际市场，千方百计扩大外贸进出口。加快“走出去”步伐，鼓励支持企业开展境外加工生产、承包工程和劳务合作，培育本土跨国公司。加快广澳港区口岸查验“一站式服务”办公楼建设，进一步改善通关环境。争取国家尽快批准建设综合保税区，推动保税区与广澳港区港联动。

　　率先建设粤台经贸合作试验区。充分运用检验检疫特殊优惠政策和支持措施，发挥对台直航航线作用，推进粤台、汕台经贸合作。全力推进粤台合作南山湾科技园、粤台物流园区、广东省海峡两岸合作南澳实验区和台湾农民创业园等载体建设，加强与台湾产业对接。以打造文化旅游品牌为重点，拓展汕台社会文化交流的深度与广度。

　　加快打通对内对外通道。规划建设连接全市六区一县的快速干线，形成全市域快速交通网络。力争南澳大桥年底合拢。加快建设汕头至揭阳高速公路汕头段和汕头城市候机楼，确保与潮汕机场同步投入使用。争取开工建设汕头至揭西、揭阳至惠来、潮州至惠州高速公路汕头段。加快建设厦深铁路进汕头站联络线，加紧规划建设厦深铁路潮阳（谷饶）站场，配套建设快速通道，确保与厦深铁路同步建成。积极做好阜鹰汕铁路梅州至汕头段、汕头港疏港铁路等一批项目的前期工作。积极引进大型码头运营商和航运龙头企业参与广澳深水港建设，完成广澳港区控制性详规和珠池港区功能调整专项论证的编制、报批工作，开工建设广澳港区防波堤工程，加快2个10万吨级集装箱泊位、10万吨级航道工程、2万吨级石化泊位前期工作。

　　（五）努力推进基本公共服务均等化

　　推动教育均衡化发展。加快普及高中阶段教育，投入8.05亿元，完成卫生学校新校区、金山中学小礐石校区等26个高中阶段学校校舍建设项目，大幅增加高中阶段学位。推进义务教育均衡发展。大力发展学前教育，恢复和兴办一批公办幼儿园，力争每个中心镇（街道）都有1所以上中心幼儿园。重视特殊教育。支持办好汕头大学、粤东高级技工学校，提高汕头职业技术学院办学水平。进一步落实中小学教师福利待遇“两相当”，妥善解决中小学代课教师转岗和解除劳动关系的问题。

　　加强医疗卫生建设。加快农村三级医疗卫生服务网络和城市社区卫生服务机构建设，实施基层卫生人才队伍建设工程，力争每千人口医生数和每千人口病床数分别达到1.35人和2.42张。加快建立基本药物制度。推进公立医院改革。继续加强疾病预防控制，防止重大传染性疾病发生流行。大力开展群众性爱国卫生运动，扎实推进农村改水改厕。

　　加快发展文化体育事业。扎实推进文化强市的各项工作。做好新华书店改革重组和市直文艺院团改革工作。加强公共文化设施建设，投入200万元，新建、改扩建10个乡镇街道综合文化站，确保全市乡镇综合文化站达标率85%以上。投入450万元，完成市博物馆新馆配套验收并对公众正式开放。建设有线数字广播电视网络、高清互动数字电视工程。继续实施广播电视村村通、农村电影放映、农家书屋等文化惠民工程。抓好潮汕文化（汕头）生态保护区的申报和建设，实施文化遗产保护工程。办好第12届市运会。加快推进社区体育健身工程，积极创建城市体育先进社区。

　　提升社会保障水平。加大社保扩面征缴力度，努力实现应保尽保。提升社会救助水平，着力提高低保补助水平。加大城乡社会福利设施建设力度，大力发展慈善事业。实施积极就业政策，加强基层就业服务平台建设，力争100%的镇（街）、80%的社区具备基本公共就业服务功能。进一步完善创业工作体系，推动创业带动就业。完善医疗救助制度，积极发展社会慈善医疗救助，力争覆盖全市所有困难家庭。

　　（六）着力提高人民生活水平

　　增加城乡居民收入。着力提高低收入劳动者工资水平，全面建立与经济发展及物价增长联动的最低工资标准调整机制。适当调整机关事业单位职工工资，加快推进事业单位实行绩效工资。逐步提高企事业离退休人员收入水平，缩小区域内公职人员收入差距和社会不同阶层收入差距，有效扩大中等收入者比重。加大对特殊群体的救助。建立健全城乡低收入困难群众社会救助体系，加大法律援助和法律救助力度。妥善解决农村“五保户”和特困户的生活困难问题。重视发展养老事业，加快构建适度普惠型社会福利服务体系，力争把低保困难家庭中年龄在70岁以上的老年人列入高龄津贴范围。重视解决优抚对象、困难群众生活保障问题。

　　着力改善人民生活环境。建立经常性严打工作机制，持续整治社会治安重点地区和突出问题，切实增强人民群众的安全感。拓宽群众利益诉求渠道，加强与上访群众的情感交流，深入开展人民调解工作，全力维护社会稳定。严格企业安全生产主体责任，强化安全生产监督管理，进一步深化重点行业领域隐患排查治理。加大安全生产执法监察力度，增强安全生产保障能力，坚决遏制较大以上事故发生。引进华润集团参与我市国有管道燃气企业的改制，整合中心城区其他燃气企业，全面推进中心城区燃气管道建设。进一步整顿和规范市场经济秩序，加强市场价格监管，切实抓好食品安全等专项整治。加强人口计生管理，提高人口素质，稳定低生育水平。继续推进殡葬改革。

　　（七）扎扎实实办好十件民生实事

　　1、做好扶贫开发“规划到户、责任到人”工作，投入7000万元，完成2511户以上贫困户脱贫任务，提前一年实现省下达的80%以上贫困户脱贫的目标。

　　2、增加就业岗位，加强就业培训，实现城镇新增就业8.3万人，安排农村劳动力参加职业技能培训2.05万人，新增转移本市农村劳动力7.6万人。

　　3、继续实施校舍安全工程，投入2.25亿元，完成各区县校安工程项目52个、18.44万平方米。

　　4、引进“全民低成本健康”工程，投入190万元，改善农村基层医疗单位检查和信息化水平，上半年在濠江、南澳14个基层医疗单位试点，下半年在澄海、潮阳、潮南18个单位开展。

　　5、扎实推进城乡社会保险。投入4.86亿元，建立商业保险与城镇居民基本医疗保险、新型农村合作医疗有机结合的城乡居民一体化医疗保险体系，力争全市基本医疗保险覆盖率达到90%以上；投入1.73亿元，推进龙湖、澄海、潮阳、潮南新型农村社会养老保险试点工作，确保今年新型农村社会养老保险制度覆盖率达到60%以上；出台解决企业未参保人员纳入企业职工基本养老保险问题的实施办法，解决城镇职工养老遗留问题。

　　6、加大社会救助力度。投入1.47亿元，将全市人年均收入低于1500元的困难家庭全部纳入低保；投入1068万元，为1000名贫困精神病患者、6岁以下残障儿童、贫困白内障患者提供免费康复救助治疗；投入270万元，资助300名贫困家庭先天性心脏病患儿接受手术治疗。

　　7、加快建设保障性住房。投入3.4亿元，筹建廉租住房1330套、经济适用住房245套、公共租赁住房2000套，新增发放租赁补贴489户，改造国有林区棚户区和国有林场危旧房29户；投入4066.5万元，完成2711户农村低收入危房户改造任务；投入250万元，为100户农村低保户修建住房。

　　8、解决城乡群众饮水安全问题。投入4000万元，解决农村10万人饮水安全问题；投入8500万元，基本完成中心城区“城中村”和涉农社区供水直抄到户、同城同价改造工程；投入1500万元，启动中心城区居民住宅小区供水直抄到户改造工程，今年完成15个多层住宅小区改造任务。

　　9、加快发展城市公共交通。投入4亿元，确保新增公共汽车300部，力争新增公共汽车800部，配套建设公交站场，恢复、增开一批公交线路，改善群众出行条件。

　　10、加强社会治安综合治理，投入820万元，增加视频监控点500个，力争今年“两抢一盗”发案率同比下降10%；投入2.2亿元，加快智能交通监控系统建设，加强交通秩序集中整治，力争今年交通事故宗数同比下降5%。

　　（八）增强政府推动科学发展能力

　　提高科学民主决策水平。认真落实人大及其常委会各项决议决定，坚持向人大及其常委会报告工作，自觉接受监督。认真接受人民政协的民主监督，主动听取各民主党派、工商联、无党派人士和各人民团体的意见建议。完善重大事项决策的规则和程序，推行行政决策过程和结果公开。加大行政决策法律审查和规范性文件管理力度，推进政府决策的科学化、民主化、法治化。

　　大力推进依法行政。加强政府立法、行政复议和行政执法监督，推进行政复议委员会试点工作，发挥行政复议解纷功能。加大清权确权力度，编制行政权力运行流程图，规范行政自由裁量权行使程序，推进行政权力公开透明运行。深入开展法制宣传教育，提高全民法律素质。综合运用调解、仲裁等法律机制，促进社会矛盾的化解。

　　加强政府执行力建设。健全行政问责机制，重点问责不作为和乱作为，做到有责必问、问责必严。加强行政监察和审计监督，规范权力运行，重点加强涉及公共利益事项的监督。建立绩效考核评估机制，尽快形成有效的激励机制，提高各级政府部门的行政效能。深入开展双拥创建活动，密切军政军民关系。切实改进机关作风，密切联系群众，问政于民，真心实意为群众多做好事实事。完善各级政府应急组织体系建设，加强各类突发事件预测预警和信息收集报送工作，提高突发事件处置能力。

　　各位代表，落实“十二五”规划的美好蓝图，推动汕头的跨越式发展，责任重大而光荣。让我们更加紧密地团结在以胡锦涛同志为总书记的党中央周围，以科学发展观统领经济社会发展全局，在市委的正确领导下，同心同德，奋力拼搏，为实现“五年大变化”，加快建设幸福汕头而努力奋斗！
